# Avian haemosporidian parasites affecting non-descript village chickens in Africa

**DOI:** 10.1007/s11250-024-04250-1

**Published:** 2025-01-20

**Authors:** Tlalemasego Matloa, Rulien Erasmus, Maphuti Betty Ledwaba, Dikeledi Petunia Malatji

**Affiliations:** 1 Department of Agriculture and Animal Health, College of Agriculture and Environmental Science, University of South Africa, Florida, South Africa; 2 Animal Production, Agricultural Research Council, Irene, South Africa

**Keywords:** Scavenging chickens, Smallholder farmers, *Haemoproteus*, *Leucocytozoon*, *Plasmodium*

## Abstract

Smallholder farmers in most of the rural areas in African countries rear non-descript village chickens for petty cash, food provision and for performing rituals. Village chicken production systems are regarded as low input- low output because the chickens receive minimum care and produce average to less eggs and meat. The chickens receive minimal biosecurity and are often left to scavenge for feed and thus exposes them to potential vector parasites that can transmit parasites such as haemoparasites. Haemosporidian parasites (Haemosporidia, Apicomplexa) are blood parasites infecting avian species, especially chickens. They are transmitted by blood sucking vectors such as biting midges, mosquitoes, black flies and louse flies. Infections are mild to severe causing reproduction, production and health losses such as decreased fertility, reduced body weight and egg production, anaemia and inflammation of vital organs such as the liver and spleen. Haemoparasites infections in chickens can be lowered through controlling vector parasites and the use of antimalarial drugs on exotic chicken breeds. The aim of this review is to characterize the avian haemosporidian parasites affecting non-descript village chickens in Africa, describing their morphology, life cycle, pathogenicity, control and prevention measures.

## Introduction

Chicken production in Africa plays an integral role in smallholder farming systems and in most developing countries, almost all families in villages, even those without land, own chickens. The majority of people in villages in African countries keep a small number of chickens for subsistence, to generate income and for various socio-cultural uses under diverse environmental, economic and cultural scenarios (FAO 2014). These systems rely on rearing scavenging chickens commonly referred to as village chickens, that are subjected to natural selective pressures. Hence, purpose driven selection and breeding are undocumented in village chickens and specific breeds are uncommon (Muchadeyi and Dzomba 2017). Although some village chickens are classified into breeds, the majority of village chickens are crossbred and remain unclassified, hence are referred to as non-descript. Smallholder farmers do not have breeding programmes and acquire village chickens from neighbours or relatives as a gift and for cultural purposes; village chickens’ classification is according to their geographical areas and observable traits (Manyelo et al. 2020). Furthermore, village chickens from the same neighbourhood breed while scavenging for feed, resulting in non-descript chicken breeds (Kumaresan et al. 2008; Malatji et al. 2016).

Village chickens play a significant role in rural households as the main source of income even though the monetary income is low. Non-cash outputs such as household meat and eggs consumption, social obligations and status, cultural purposes and the use of manure as fertilizers are non-financial returns provided by village chickens (Mapiye et al. 2008). Both eggs and white meat provided by village chickens are rich in protein and in Southern Africa, high consumption of animal protein in rural communities mainly comes from village chicken meat and eggs (Mtileni et al. 2012). Production of village chickens alleviates poverty in developing countries and rural communities, also empowering women and children in female headed households (Desta 2021). Village chicken products provide people living with HIV/AIDS, people living with disabilities, pregnant women, nursing mothers and children with essential micronutrients (Moreki et al. 2010). Elderly people use village chickens as a token of appreciation for services rendered, building of friendships and for cultural ceremonies (Malatji et al. 2016).

While village chickens require few inputs, they are reared in challenging environments constrained by disease, scarcity of feed and predation leading to low productivity and high mortality (Bettridge et al. 2018). Chickens are susceptible to several types of parasitic infections, of which haemoparasitic infections are considered the most common and significant (Lawal et al. 2016). Haemosporidian parasites are a group of blood endoparasites that affect various avian species, with genera *Haemoproteus*, *Leucocytozoon* and *Plasmodium* most commonly reported in chickens (Tembe et al. 2023). The significant economic importance of blood parasites in chickens is related to increased mortalities, retardation of growth, reproductive failure and decreased immunity (El-Ghany 2023). However, the majority of research on diseases affecting poultry has focused on viral infections such as avian influenza, infectious bursal disease and Newcastle disease, with limited investigations on haemoparasites (Valkiūnas et al. 2005; Sabuni et al. 2011). Investigations in Africa, and specifically in non-descript village chickens, are also neglected. Therefore, the main aim of this review was to search and explore the existing literature to determine the haemosporidian parasites prevalent in non-descript village chickens in African countries, and describe their morphology, life cycle, pathogenicity, as well as control and prevention measures. Public databases such as Google Scholar, PubMed, Science Direct and Web of Science were targeted.

## Village chicken production systems

The purpose for keeping village chickens usually depends on access to markets, with food security being of higher priority in remote villages, whereas villages with access to rural markets place emphasis on income generation (FAO 2014). Majority of smallholder farmers prefer to rear chickens over other livestock species since they have short production cycles, are able to reproduce under low production inputs and minimum care. Village chickens are considered as one of the most adaptable domestic animals to survive in harsh environments because of their adaptation traits, such as excellent mothering ability, low maintenance requirements and dark plumage colour acting as camouflage, which enable them to adapt to local environmental conditions (Gunya et al. 2020). Moreover, village chickens have a natural strong instinct to predict danger and respond quickly; and they are able to fly and run from predators as compared to exotic breeds (Manyelo et al. 2020).

Village chickens are reared under extensive management systems, classified according to FAO (2014): i) small extensive scavenging, with one to five adult birds, limited provision of poultry housing and little access to veterinary services, or ii) extensive scavenging, with five to fifty adult birds, some provision of poultry housing and limited access to veterinary services. These extensive systems are characterized by improper feeding, low input and output, inadequate shelter and small flocks (Assefa et al. 2019). Both of these systems experience high mortality rates (> 70%) and do not have access to supplementary feeding, although the role of kitchen food scraps as supplementary feeding are not clear (FAO 2014; Desta 2021).

Village chickens are kept under suboptimal conditions having no separate shelter except for basic night enclosure and smallholder farmers depend on cheap and freely available resources for flock maintenance (Desta and Wakeyo 2013). Where shelter is provided, it is usually not well planned or constructed and barely provides minimum protection (Melesse 2014). The construction of shelters are made from locally available materials such as wooden poles, bricks, mud and tree branches. Soil, grass, straw, wood and wood shavings are used for flooring; and iron sheets are used for roofing (Muchadeyi et al. 2004; Mapiye et al. 2008). In north-west Ethiopia, smallholder farmers use hand woven baskets, bamboo cages, or usually the main house and a section in the kitchen as night shelter for their chickens (Mogesse 2007).

In some African villages, chickens are not confined during day-time and it is common practice for village chickens to scavenge for feed; a characteristic feature of indigenous non-descript chicken production systems (Melesse 2014). Usually, the chickens scavenge for feed and water around the homestead and nearby surroundings (open spaces, nearest dumping sites and cattle kraals) (Melesse 2014). These chickens feed on insects, worms, pastures, seeds, cultivated field gleanings, minerals from the soil and household leftovers (Idowu et al. 2021). The amount of feed scavenged during the day rarely meets the daily nutritional requirements of the chickens for maintenance and optimal production, hence leading to decreased egg production and minimum carcass weight. Some smallholder farmers might provide supplementation to their hens and cocks with crops grown in their backyards. Leguminous plant leaves, cassava and sweat potatoes leaves; *Moringa* species and *Leucaena* species tree leaves serve as good sources of protein (Melesse 2014).

Domestic market of village chickens is eco-friendly with less competition for scarce land resources (Assefa 2019). Marketing of village chickens is usually done through word of mouth and door to door sales. The market system involves a long chain of actors and middlemen; thus, creating job opportunities such as transporting eggs and chickens to the market place and to wholesalers by the middlemen involved (Desta 2021). However, the disadvantage of marketing with middlemen is that they get an average earning of up to 65% of the profit, which is more than what the smallholder farmer receives; thus, a highly strategic plan needs to be implemented to benefit both farmers and traders (Mlozi et al. 2003; Asem-Bansah et al. 2012; Desta 2021). In some African countries such as Ethiopia, women stay at home for reasons such as men dominating the marketplaces, men being involved in rearing chickens and taking decisions on the sales (Assefa 2019). Customers prefer to buy live chickens in order to inspect their health status and pricing of chickens is determined by sex, season, age, plumage colour, health status and meatiness. Informal marketplaces and relationships negatively affect the pricing of village chickens, as neighbours and relatives buy at a cheaper price as compared to other customers (Desta 2021).

## Diseases and parasites as the main challenges of rearing village chickens

A major threat to rearing village chickens extensively is the increased occurrence of diseases. Minimum biosecurity, less veterinary care and pharmaceuticals exposes village chickens to a greater risk of being infected by infectious diseases such as Newcastle disease (ND), coccidiosis, fowl pox, infectious bursal disease (IBD) and being in contact with vector parasites transmitting avian haemosporidian parasites. Smallholder farmers have limited information regarding biosecurity and disease prevention and control measures (Wong et al. 2017; Enahoro et al. 2021). In most of the rural areas in Africa, smallholder farmers farming with village chickens receive less extension services, and where services exist, they are mostly focused on ruminants and crop production (Wong et al. 2017). In addition, smallholder farmers lack knowledge about the emergence of diseases, their control and prevention measures; and they also lack vaccination skills (Melesse 2014). With the existing parasitic diseases, haemoparasitic infections are highly prevalent and heavy parasitic infections can lead to susceptibility to diseases, pain and discomfort affecting daily normal activities, bleeding and death may also occur (Opara et al. 2014; Manyelo et al. 2020).

Scavenging for feed and water exposes village chickens to diseases and parasites co-existing in scavenging environments (Opara et al. 2014). Compared to adults, chicks and juveniles are more susceptible to diseases because of their underdeveloped immune system (Idowu et al. 2021). Several parasites, such as avian malaria caused by *Plasmodium* infections, can result in mortalities and suppression of the chicken’s immune system (Opara et al. 2014). A study conducted in South-Eastern Nigeria reported that infections with *Leucocytozoon* species cause *Leucocytozoonosis* resulting in weakness, dyspnea, listless, inappetence and mortalities in the first 24 h in chicks and juveniles. In adult chickens, the clinical symptoms are less severe, and mortality is rare. Additionally, *Leucocytozoon* infections cause decreased egg production as they affect the hens’ reproductive organs such as the ovaries and the oviduct (Nguyen 2019).

Flock sizes of African non-descript village chickens are very small comprising of an average number of 5 to 20 chickens per household and one of the reasons for small flock sizes is the presence of diseases and parasites. The flock structures consist of adult chickens, juveniles and chicks; with the juvenile group making 60% of the flock. The flock mating ratio is usually 1 cock: 5 hens, however, two studies investigating the village chicken population in Ethiopia reported a mating ratio of 1 cock: 1.8 female (Desta 2021). Three studies conducted in Nigeria (*n* = 2) (Lawal et al. 2016, 2021a) and Kenya (*n* = 1) (Sabuni et al. 2011) (Table 1) recorded a higher prevalence of the haemoparasites in males as compared to females. Lawal et al. (2016, 2021a) indicated that this might be caused by the phenotype of the male’s head having a bigger comb and wattle that are supplied with blood vessels attracting parasites vectors for feeding on a blood meal. The fully developed comb and wattle of adult chickens plays a role in the attraction of arthropod vectors.

**Table 1:** The prevalence of haemosporidian parasites in non-descript village chickens according to age and sex in different African countries and regions

Country	Region	Haemosoridian parasites species	Total Prevalence (%)	Age	Prevalence per age (%)	Sex	Prevalence per gender (%)	References
Nigeria	Maiduguru	*Haemoproteus* and/or *Plasmodium* species *Haemoproteus* species *Plasmoduim* species	17.6 50.9 29.4	NS	NS	Male Female	20.5 11.5	Lawal et al. 2016
Nigeria	Dukku, Gombe Abbah and Hashidu	*Plasmodium* and/or *Haemoproteus* *Plasmodium* *Haemoproteus*	1.9 15.9 4.1	Adults Grower	16.5 5.4	Male Female	14.3 7.6	Lawal et al. 2021a
Kenya	Embu District and Mbeere District	*Plasmodium gallinaceum* *Leucocytozoon schoutedeni* *Haemoproteus* specie	53.7 52.1 3.5	Adults Grower Chicks	81.3 83.3 72.9	Male Female	83.3 75.0	Sabuni et al. 2011

*NS:* Not specified

In Nigerian and Bishofu chickens, adults had more infections as compared to chicks and juveniles (Lawal et al. 2021a; Etisa et al. 2017) since adult chickens travel distances to scavenge for food and looking for their mates and this exposes them to potential parasites vectors while younger chickens are always closer to their brooding space. Lawal et al. (2021b) reported that in Nigerian village chickens, the *Leucocytozoon*, *Haemoproteus* and *Plasmodium* infections were high during the wet season and low during the dry season. Okanga and Cumming (2013) (South Africa) and Igbokwe et al. (2008) (Nigeria) further reported that *Plasmodium* single infections were more prevalent during the rainy season, which coincides with increased breeding activity of parasite vectors.

## Classification and morphology of haemosporidian parasites in village chickens

Avian haemosporidian parasites are a group of blood endoparasites infecting avian species, both domestic and wild. Haemosporidian parasites are unicellular eukaryotic parasites detected using multiple diagnostic methods, of which traditional methods and molecular technologies are most commonly used. Traditional methods identify haemosporidian parasites’ morphology on blood smears using a microscope (Ciloglu et al. 2016). Polymerase Chain Reaction (PCR) is a molecular technology that can determine parasite density and identifying the parasites to species level through sequencing the amplified DNA (Bensch et al. 2009; Ibrahim and Al-Rubaie 2020).

Haemosporidian parasites are vector-borne and they develop in stages in the infected hosts tissues and blood (El-Ghany 2023). More than 277 haemosporidian parasites have been recognised among 23 host orders and molecular studies identified more than 2 000 unique genetic haplotypes (Ciloglu et al. 2016). Avian haemosporidian parasites belong to the phylum Apicomplexa and the genera *Plasmodium, Leucocytozoon*, *Haemoproteus*, *Trypanosoma*, *Fallisia*, *Aegyptinella* and *Microfiliaria*. These parasites genera are spread world-wide except for genera *Haemoproteus* which does not occur in Antarctica due to the absence of vectors that transmit the parasite (Boonchuay et al. 2023). The most prevalent genera recorded in village chickens in Africa are *Leucocytozoon*, *Haemoproteus* and *Plasmodium*, and their transmissions are through vectors such as mosquitoes, black flies, lice, biting midges and fleas (Ogbaje et al. 2019). In village chickens, the most common species recognised world-wide are two species of the genera *Plasmodium* (*P. gallineceum* and *P. juxtanucleare*), three *Leucocytozoon* species (*L. sabrazesi*, *L. schouteni* and *L. caulleryi)* (Valkiūnas 2005; Agbemelo-Tsomafo et al. 2023), two *Haemoproteus* species (*H. pratasi* and *H. sacchorovi*), three *Trypanosoma* species (*T. calmetti*, *T. gallinarum* and *T. numidae*) (Sehgal et al. 2006) and one Aegyptinella specie (*A. pullorum*) (Suleiman 2012).

### Leucocytozoon

The *Leucocytozoon* genus has more than 100 species infecting over 100 avian species. *Leucocytozoon* gametocytes have indistinguishable morphology. Gametocytes are round to oval, overlapping in form and size in most of the species (Valkiūnas and Iezhova 2023). The size of mature gametocytes is 10–15 μm and the host cell size is almost 20 μm in diameter (Adamu 2017). The unique life cycle of *Leucocytozoon* parasites, which infects not only erythrocytes but also leucocytes makes morphological identification difficult (Valkiūnas 2005). Even though about 45 morphologically distinct species of *Leucocytozoon* parasites have been described for causing leucocytozoonosis disease in wild and domestic birds (Valkiūnas and Iezhova 2023), only a few of these species have been described in poultry. *Leucocytozoon* species were classified according to the avian species in which they were detected, i.e. *L. sabrazesi* in fowls, *L. simon* and *L. caulleryi* in anseriformes, *L. smithi* in turkeys, *L. ziemanni* in owls, *L. marchouxi* in columbiformes and *L. toddi* in falconiformes (Hasson 2015). In chickens, *L. caulleryi* is highly pathogenic causing reduced egg production and soft-shelled eggs and fatal hemorrhagic diseases in pullets and cockerels (Nakamura 2022).

### Haemoproteus

*Haemoproteus* species are largely distributed across Africa and they are prevalent in chickens with its symptoms being mild to asymptomatic (Donovan et al. 2008). Over 150 species of this genus have been described and the morphological characteristics of the *Haemoproteus* species are less distinctive and sometimes the identification of species is not reliable at the sporogonic cycle and the tissue stage in vectors (Valkiūnas et al. 2019). *Haemoproteus* species do not undergo erythrocytic merogony and have fewer distinctive characters at species level. The gametocytes produce pigment granules which stains golden brown to black colour on the blood smears when viewed under polarized or dark-field light microscope.

### Plasmodium

Haemosporidian infections in poultry species causes diseases, however, in most cases the *Plasmodium* species are the only species specified as avian malaria parasites (Ibrahim and Al-Rubaie 2020). The established avian *Plasmodium* species were dominant in birds living in tropical and subtropical countries and in birds that are adapted to living in warmer environments as reported by Valkiūnas and Iezhova (2018). More than 65 *Plasmodium* species have been established in more than 1000 avian host species and the most life threating *Plasmodium* species can cause up to 90% flock mortalities (Ibrahim and Al-Rubaie 2020; Panhwer et al. 2016). *Plasmodium* infections are not host specific and are transmitted by numerous vectors (El-Ghany 2023). Different mosquito vectors transmit different *Plasmodium* species: *P. juxtanucleare* is transmitted by various species of *Culex* mosquitoes (common house mosquitoes); *P. gallinaceum* and *P. juxtanucleare* are transmitted by Diptera: Culicidae mosquitoes (midge); and *P. gallinaceum* being transmitted by *Mansonia crassipes* (mosquito) and *Culex quinquefasciatus* (southern house mosquitoes) (Boonchuay et al. 2023).

*Plasmodium gallinaceum* gametocytes and meronts are irregular, round or oval in shape. The host cell’s nucleus is hardly expelled during infection and can be replaced by the parasite. An average of 16–20 merozoites are produced by a single meront. The shape of *P. juxtanucleare* schizonts is irregular, ovoid or round and usually smaller than those of *P. gallinaceum* and the shape of the gametocytes is round, ovoid or slightly elongated. Meronts produce an average of 3–5 merozoites (Permin and Hansen 1998).

## Life cycle of Haemosporidian parasites

**Fig. 1:**
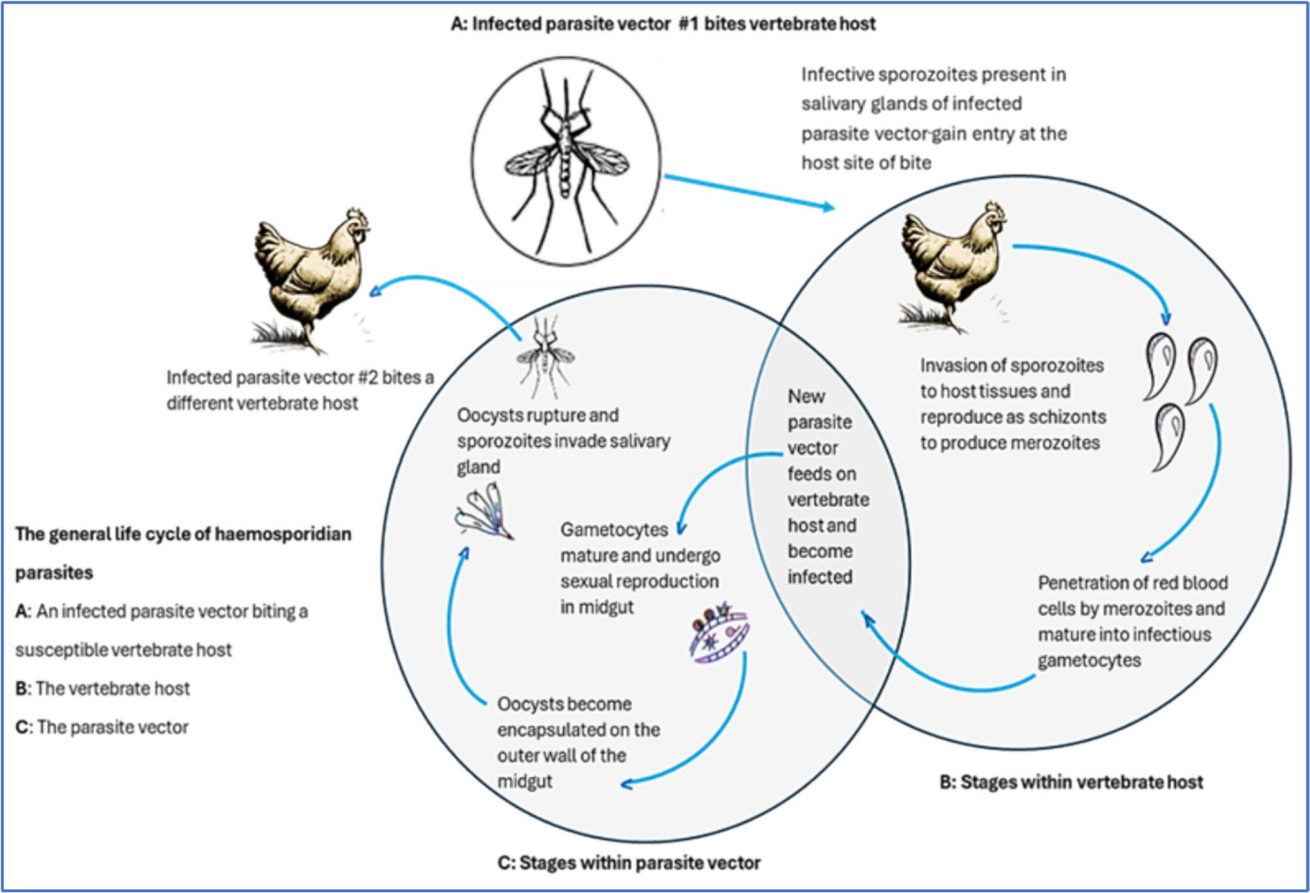
The life cycle of haemosporidian parasites starting with an infected parasite vector (A) biting the vertebrate host #1, followed by separate stages (infectious and developmental) within the vertebrate host (B) and the parasite vector (C)

The life cycle of haemosporidian parasites (*Leucocytozoon*, *Haemoproteus* and *Plasmodium*) are similar, involving asexual reproduction in both the parasite vector and the vertebrate host (Kvasager 2015). Parasitic infections are transmitted from one host to another by parasite vectors through a bite of an infected blood meal (Fig. 1: #1). During feeding, sporozoites invade the vertebrate host through the vector parasites salivary gland secretions (Fig. 1A). Post entry to the host’s bloodstream, the sporozoites infect the host tissue cells (liver and spleen) and undergo asexual reproduction (schizogony) leading to the formation of meronts (Kvasager 2015). The single celled merozoites are then released to the circulating blood cells when the tissue cells rupture of which they may continue schizogony or develop into micro (male) or macro (female) gametes (gametogony) (Fig. 1B).

The flagellated microgametes fertilize macrogametes in the parasite vector’s midgut forming an ookinete. Ookinetes migrate to the peripheral of the blood meal and invade the epithelial cells of the midgut forming an oocyst. The developed oocyst then undergoes asexual reproduction producing elongated sporozoites (Fig. 1C). After release, the sporozoites further invade the salivary glands of the vector and are ready to infect new avian vertebrate hosts and the life cycle of the parasites is ready to be repeated (Valkiūnas 2005; Bell 2016) (Fig. 1: #2).

### *Leucozytozoon* life cycle

*Leucocytozoon* infections are transmitted by Simulid flies while other *Leucocytozoon* species such as *L. caulleryi* are transmitted by biting midges. After a blood meal, the sporozoites travel to the parenchymal cells to start merogony. First phase of merogony takes place involving the sporozoites developing into schizonts followed by rupture of mature tissue meronts releasing the merozoites. The released merozoites enter the mature and immature blood cells and later develop into gametocytes (Walther et al. 2016). In avian species infected by *Leucocytozoon*, the exoerythrocytic merogony takes place in the macrophages, multiple endothelial cells and in the parenchymal cells. During the second phase of merogony, formation of megalomeronts takes place in the spleen and lymph-nodes. The megalomeronts release merozoites forming large fusion gametocytes (Valkiūnas and Iezhova 2023), however, the maturity of the gametocytes in the circulating blood cells (erythrocytes, erythroblasts and mononuclear cells) does not have the hemozoin.

### *Haemoproteus* life cycle

Biting midge and hippoboscid flies are vector parasites transmitting *Haemoproteus* sporozoites (Ilgūnas et al. 2019). In *Haemoproteus* species the merogony phase takes place outside red blood cells and in most cases, the meronts are established in the lungs and much less in the skeletal muscle, liver, spleen and kidneys (Atkinson et al. 1988). However, in other *Haemoproteus* species such as the *H. meleagridis,* the development of megalomeronts takes place in the endothelial cells and myofibroblasts (Valkiunas 2005). Prior to the expression of the gametocytes, exoerythrocytic meronts are multigenerational and the first generation develops inside the myofibroblasts and capillaries endothelium. This development is completed five days post infection. The production of meronts and megalomeronts in the spleen and skeletal muscle which produce merozoites take place, invading red blood cells inhibiting the production of gametocytes in secondary generation. Secondary generation takes place 17 days post infection (Valkiūnas 2005; Bell 2016).

### *Plasmodium* life cycle

Vector parasites transmitting *Plasmodium* are blood sucking infected female mosquitoes (Bell 2016). Unlike *Leucocytozoon* and *Haemoproteus*, the *Plasmodium* life cycle includes merogony, taking place in both erythrocytic and exoerythrocytic cells (Atkinson et al. 1988; Valkiūnas 2005; Bell 2016; Valkiūnas and Iezhova 2018). The development of the erythrocytic meronts takes place within the red blood cells and the development of the gametocytes in the mature erythrocytes. Exoerythrocytes merogony takes place in the endothelial cells. Infected female mosquitos introduce the non-infectious exoerythrocytic meronts (crytozoites) which develop in the reticular cells such as the skin and spleen (Atkinson et al. 1988; Frevert et al. 2008). Merozoites growing in the crytozoites cause second generation of primary exoerythrocytic meronts (metacrytozoites) that are developing in macrophages of multiple organs. Metacrytozoites meronts are infectious, therefore, they reinfect the macrophages for the continuation of primary exoerythrocytic merogony, infect erythrocytes to start erythrocytic stages and lastly infect the endothelial cells to begin secondary exoerythrocytic merogony (Valkiūnas 2005). Formation of phanerozoites and release of merozoites that are able to infect blood cells take place. The released merozoites proceed with exoerythrocytic merogony or erythrocytic merogony. The gametocytes get ingested by mosquitoes from infected avian species forming gametes followed by fertilization and development of ookinetes (Bell 2016).

## Pathogenicity

### *Leucocytozoon*

#### Tissue stage

Laboratory studies targeting the blood parasites of chickens and other poultry species such as ducks, geese and turkeys as well as post-mortem studies on wild birds have shown that *Leucocytozoon* infections can result in pathological effects such as schizogony, severe anaemia as well as the congestion of the lungs, pneumonia and the blockage of the alveolar capillaries that may be caused by the circulating gametocytes (Maley and Desser 1977; Atkinson and Van Riper 1991). Post introduction of schizonts to the vertebrate blood stream, the first generation invades multiple organs causing the sinusoids to congest and dilate. In chickens infected by other *Leucocytozoon* species such as *L. caulleryi*, the released merozoites are absorbed by phagocytic cells in vital organs such as the liver, heart, brain, spleen and the lungs which tend to grow into megaloschizonts (Atkinson and Van Riper 1991; Zhao et al. 2015). The vertebrate host’s immune response towards inflammation on the infected bird species takes place during the megaloschizogony phase where the parasites are enclosed by fibrotic tissue and surrounded by the red blood cells, macrophages, heterophils and plasma cells mixed inflammatory infiltrates (Zhao et al. 2015). The megaloschizonts in the affected tissue may become calcified or die (Atkinson and Van Riper 1991).

#### Blood stage

Severe infections of *Leucocytozoon* destroy the host’s blood cells causing anaemia. The destruction takes place in the spleen where infected blood cells destroy the cells of the reticulo-endothelial system. Unparasitesized erythrocytes are also destroyed by putative anti-erythrocyte factors emerging from the serum in severe infections (Atkinson and Van Riper 1991; Zhao et al. 2015; Nakamura 2022).

### *Haemoproteus*

#### Tissue stage

Studies on the pathogenicity of *Haemoproteus* are very limited, only studies on naturally infected bird species and *H. columbae* infections in pigeons have been conducted (Cepeda et al. 2019). Asexual schizogony of the *H. columbae* takes place in the epithelial cells of the lungs in heavily infected pigeons where symptoms such as pneumonia and congestion can be noticed. It takes 21–32 days for the *H. columbae* to cause pathological changes visible through clinical symptoms such as ruffled feathers, inability to move and depression. It is believed that other *Haemoproteus* species traditionally go through asexual schizogony in the lung tissue causing minor pathological changes. In parakeets naturally infected with *H. bandai* and in domestic turkey’s experimentally infected *H. meleagridis* in South-East, it was reported that there were developments in the myofibroblasts and capillary endothelial cells in the skeletal muscles (Atkinson et al. 1988). There were two schizogony generations as reported by Atkinson et al. 1986 which prior merozoites invaded red blood cells and matured into gametocytes and they resulted into death of the surrounding tissue and lameness in the infected birds (Atkinson et al. 1986). The necrosis of the surrounding muscles progressed to be calcified as the infections proceeded (Atkinson et al. 1986, 1988).

#### Blood stage

*Haemoproteus* infections cause a discolouration of the spleen and anaemia. The *Haemoproteus* as compared to *Plasmodium* parasites do not reproduce in the blood, thus the infections on a new host through direct feeding on blood is unlikely to occur (Cepeda et al. 2019).

### *Plasmodium*

#### Tissue stage

*Plasmodium* infections may prevent blood flow to the brain capillaries and spleen by phanerozoites or alternatively cause severe anaemia in the hematopoietic system cells. The blockage result in the cells of the affected organs dying off due to anoxia on the tissues that surrounds the meronts. The development of infiltrates takes place followed by oedema and dying of the tissues surrounding the meronts. Mortalities from cerebral paralysis can occur and indistinguishable pathological changes are attributes caused by *Plasmodium* species such as *P. gallineceum*, *P. durae*, *P. lophurae*, *P. fallox* and *P. cathemerium* (Valkiūnas 2005).

#### Blood stage

Infections with *Plasmodium* lead to a destruction of erythrocytes linked with erythrocytic schizogony. The erythrocytes are destroyed when the infected red blood cells rupture in time of release of the meroites by the mature intracellular schizonts and elimination of the infected red blood cells by the reticulo-endothelial system cells. (Atkinson and Van Riper 1991).The blood plasma pH decreases and protein concentration increases during heavy parasitemia, leading to a decline in the oxygen binding capacity of haemoglobin (Valkiūnas 2005).

### Control and prevention

It is recommended that investigations of haemosporidian parasites using molecular technologies in village chickens of all sexes and age should be conducted in African countries where such studies have not been conducted in order to identify the prevalent parasite species and implement control strategies. This is significant since the majority of the village chickens of all ages and both sexes run together in the flock, therefore chances of infections could be increased. The investigations should not be limited to the seasons as different vector parasites are present in different seasons (wet and dry season). Agricultural extension services, including training and workshops to smallholder farmers regarding the parasites, parasites diseases, biosecurity and the use of pharmaceutical products should also be provided.

### The use of antimalarial drugs

Antimalarial drugs do not clear the haemoparasitic infections but they lower the parasitemia. Different drugs are used to control infections from different parasitic diseases. However, for *Haemoproteus* species, no antimalarial drugs have been established for commercial use and several drugs have been tested on owls and pigeons (Van Wettere 2023a). *Leucocytozoon* infections are prevented with pyrimethamine (1 ppm) and sulfadimethoxine (10 ppm) medicated on feed. These mentioned medications are effective on preventing *L. caulleryi* infections (Van Wettere 2023b). For *Plasmodium* infections, antimalarial drugs are not commercially available for flock treatment but a combined mixture of sulfaquinoxaline and trimethoprim added to the chickens feed is effective on chickens infected with *P. gallinaceum*. Alternatively, chloroquine (50 mg/kg) has been experimentally tested to treat *P. juxtanucleare* infections in exotic breed such as Leghorn (Van Wettere 2023c), however, the efficacy of the use antimalarial drugs on village chickens reared by smallholder farmers has not been reported.

### The use of ethno-veterinary medicine

Ethno-veterinary medicine is widely used by resource poor smallholder farmers that have little to no knowledge about chemical remedies and medications, have limited access to veterinary services and have lack of training and skills of treating parasites and diseases (Gobvu et al. 2023; Sharma et al. 2022). Some of the remedies used are jeyes fluid, potassium permanganate, *sekgophana* (Aloe vera) and smoke in the chickens houses. *Thamnosma rhodesica* leaves are also distributed inside the chickens’ enclosures to repel parasites (Moreki et al. 2010). However, the safety and efficacy of most of these remedies are not known as they have not been tested and validated.

### Managing parasite vectors

The presence of arthropod vectors in the ecosystem poses a higher risk of transmission of haemosporidian parasites to chickens, therefore, these vectors can be managed by spraying poultry houses with insecticides, using traps especially at night to collect the insects, and eliminating stagnant water that can act as a breeding environment for mosquitoes near poultry houses (Msotte and Cardona 2009; Van Wettere 2023c).

## Conclusion

Haemosporidian parasites are blood parasites widely distributed in Africa affecting non-descript village chickens that are mostly reared extensively and semi-extensively. Due to the scavenging behaviour of non-descript village chickens, they are exposed to environments that harbour potential parasite vectors. Common clinical symptoms observed in village chickens heavily infected by haemoparasites are anaemia, weight loss, infertility and death. Diagnostic methods used to detect haemosporidian parasites are microscopic, for the morphology, and PCR to identify the parasites lineages to species level. The life cycles of *Leucocytozoon*, *Haemoproteus* and *Plasmodium* are similar on asexual reproduction in the parasite vector and host, and differ in the location where asexual merogony takes place. In *Leucocytzoon* and *Haemoproteus*, merogony takes place outside the erythrocytes, and in *Plasmodium*, merogony takes place in the erythro and exoerythrocytic cells with the gametocytes and meronts being present in the erythrocytes. The morphology and size of the haemosporidian parasites varies from genus to genus.

## Data Availability

Not applicable.
